# Predictors of 90-Day Re-hospitalization in Heart Failure: Insights From a Prospective Observational Study in a Tertiary Care Setting

**DOI:** 10.7759/cureus.98187

**Published:** 2025-11-30

**Authors:** Muhammad Abubakar, Arva Zahid, Tasnia Hoque, Hamritha Manoharan, Omar Naeem, Irshad Muhammad, Muhammad Jamil, Shah Zaib Bhindar, Muhammad Ayoob Memon, Fnu Shahzeen, Muhammad Irfan Jamil, Adeel Ahmed

**Affiliations:** 1 Cardiology, University Hospitals Birmingham NHS Foundation Trust, Birmingham, GBR; 2 Cardiology, Multan Institute of Cardiology, Multan, PAK; 3 Cardiology, Shaikh Zayed Hospital, Lahore, PAK; 4 Internal Medicine, University Hospitals Birmingham NHS Foundation Trust, Birmingham, GBR; 5 Internal Medicine, King Edward Medical University, Lahore, PAK; 6 Internal Medicine, Medical Teaching Institution (MTI) Khyber Teaching Hospital, Peshawar, PAK; 7 Internal Medicine, Nishtar Medical University, Multan, PAK; 8 General Medicine, Umeed Health Care, Lahore, PAK; 9 Internal Medicine, Jinnah Sindh Medical University, Karachi, PAK; 10 Nephrology, Lahore General Hospital, Lahore, PAK; 11 Anesthesiology and Critical Care, King Edward Medical University, Lahore, PAK

**Keywords:** chronic kidney disease, heart failure, medication adherence, predictors, re-hospitalization

## Abstract

Background: Heart failure (HF) remains a leading cause of recurrent hospital readmissions, particularly during the early post-discharge period (first 90 days). The present study aimed to evaluate sociodemographic, clinical, biochemical, and follow-up determinants of 90-day unplanned readmission among adults admitted with HF at a tertiary cardiac center.

Methods: A prospective observational study was conducted among adults aged ≥18 years with clinically and echocardiographically confirmed HF. Baseline demographics, clinical characteristics, comorbidities, laboratory parameters, medications, and functional status were documented. Participants were followed for 90 days after discharge to assess readmission. Both single-variable and multivariable logistic regression techniques were utilized, and outcomes were summarized in terms of adjusted odds ratios along with 95% confidence intervals (CI).

Results: Of 300 patients, 270 (90%) completed follow-up, and 78 of these (28.9%) were readmitted within 90 days; 30 patients (10%) were lost to follow-up. Compared with those not readmitted, patients who were readmitted were older and predominantly from lower socioeconomic groups (p < 0.05). Readmission was more frequent among those with New York Heart Association (NYHA) Class III symptoms, chronic kidney disease (CKD), and anemia (all p ≤ 0.001). They also had lower renal function and serum sodium with markedly higher N-terminal pro-B-type natriuretic peptide (NT-proBNP) levels (p < 0.001). Failure to adhere to the treatment regimen (48.7% vs 21.4%; p < 0.001) and delayed post-discharge review within seven days (57.7% vs 42.3%; p = 0.003) were found to substantially increase the likelihood of readmission. Multivariable logistic regression demonstrated that patients categorized as NYHA Class III had a significantly greater likelihood of readmission (adjusted odds ratio (AOR) = 2.18; 95% confidence interval (CI): 1.15-4.13; p = 0.017). CKD also showed an independent relationship with readmission (AOR = 2.34; 95% CI: 1.02-5.37; p = 0.044). Each 1000 pg/mL increase in NT-proBNP concentration was associated with a 21% rise in the odds of readmission (per 1000 pg/mL; AOR = 1.21; 95% CI: 1.07-1.37; p = 0.002). Furthermore, individuals not receiving angiotensin-converting enzyme inhibitors or angiotensin receptor blockers exhibited higher readmission risk (AOR = 2.87; 95% CI: 1.38-5.97; p = 0.005). Suboptimal medication compliance (AOR = 2.95; 95% CI: 1.52-5.72; p = 0.001) and lack of timely post-discharge follow-up (AOR = 1.98; 95% CI: 1.05-3.73; p = 0.034) were also significant predictors.

Conclusion: Ninety-day readmission in HF was frequent and independently associated with advanced functional class, renal dysfunction, elevated NT-proBNP, lack of angiotensin-converting enzyme inhibitor or angiotensin receptor blocker therapy, poor adherence, and delayed post-discharge review. Targeted discharge education, adherence support, optimization of renin-angiotensin system blockade, and early clinic review may reduce preventable readmissions.

## Introduction

Heart failure (HF) is a major health concern to populations and a predominant source of hospitalization and re-hospitalization especially in older adults. It is known to impact more than 64 million people worldwide and leads to substantial morbidity, mortality, and healthcare expenditure [1]. The global estimated cost of the HF care service per year was above 100 billion dollars with frequent hospitalization being the major contributor. Financial burden is even greater in developing nations because there are a lack of healthcare infrastructure, limited access to specialized cardiac care, and insufficient post-discharge support mechanisms [2].

According to the American Heart Association, it is estimated that approximately 5.7 million Americans are living with HF, which translates to approximately one million hospitalizations annually [3]. Globally, over 50% of people hospitalized with HF have repeated hospitalization within six months of discharge, which has a tendency to both identify the chronicity of the disease and suggest shortcomings in current management customs [4]. Repeat hospitalization does not only fail to impose the financial burden on the healthcare systems but also cause significant physical and psychological trauma to both patients and their families [5].

The persistent impairment of the myocardium and the neurohormonal system activation have a key role in the recurring decompensations of HF patients. These mechanisms facilitate ventricular remodeling and predetermine the recurrence of exacerbations. Previous literature reported readmission rates ranging between 18% and 32% within 30 days, 21% and 47% within 90 days, and up to 50% within one year of discharge across diverse populations and healthcare settings [6-8]. The dissimilarity in the prevalence of readmission indicates the complexity of disease and disparities in health services. The following clinical factors involved in frequent admissions include advancing age, low left ventricular ejection fraction, kidney weakness, anemia, and comorbidities (like diabetes or hypertension). Furthermore, low compliance with treatment, lack of early awareness of symptom escalation, and inappropriate use of drugs recommended by the guidelines to control hypertension, such as angiotensin-converting enzyme inhibitors (ACEi), angiotensin receptor blockers (ARBs), beta-blockers, and mineralocorticoid antagonists, contribute significantly to risk [9-11]. The initial weeks following hospital discharge, frequently described as the vulnerable window, remain critical because of residual congestion and incomplete optimization of therapy [5,12,13]. Socioeconomic and health system factors substantially influence HF readmission rates. In developing countries such as Pakistan, hypertension, rheumatic heart disease, and cardiomyopathies are the major causes of HF, and ischemic disease of the heart is more prevalent in developed countries [10,11].

Financial constraints, insufficient counseling of the patient, medication expenses, and lack of follow-up care are some factors that lead to repeated hospitalization. Even though there is considerable research on it worldwide, little is known about South Asian populations. The general management of disease is constantly evolving with the changing lifestyle trends, changes in healthcare practices, medical technology, and the attitude of patients towards illness. Thus, there is a need to identify patient-level demographic, clinical, and service factors that contribute substantially to the re-hospitalization of HF patients. The problems of early readmissions can be greatly avoided and usually indicate the lapse of discharge planning, outpatient monitoring, and patient education. The purpose of conducting this study was to identify demographic, clinical, and service predictors of 90-day unplanned re-hospitalization to implement specific interventions to help reduce patient outcomes.

## Materials and methods

The prospective longitudinal observational study was carried out at the Cardiology Department, Ch Pervaiz Elahi Institute of Cardiology in Multan, Pakistan, and was approved by the Department of Academic Affairs Ethical Committee (approval number: 152; date 05/01/2024). All patients gave written informed consent before enrollment. The data collection was over a period of 12 months (January to December 2024), and all the enrolled patients were tracked within 90 days after discharge to observe hospital readmission.

The recruitment of the patients was done using a consecutive sampling technique in which all qualifying individuals were successively recruited until they were enough to reach the required number. The decision to use the sample was informed by the results of the previous data that indicated a re-hospitalization/readmission rate of 23.6% in patients with HF [14]. Using the events-per-variable principle (10 events per six covariates), the 60 re-hospitalization events were estimated as the minimum. The final target population was estimated at 300 individuals with an adjustment for the potential follow-up loss.

The study population consisted of patients aged 18 years and older who were hospitalized with HF and diagnosed according to the American College of Cardiology/American Heart Association (ACC/AHA) 2022 criteria, which include clinical assessment, echocardiographic findings, and relevant laboratory or imaging evidence [15]. All diagnoses were confirmed by the attending consultant cardiologist.

The data collection procedure was further broken down into two stages. The first stage entailed gathering of information on the pre-discharge day, after the establishment of clinical stabilization. The demographic information, clinical observations, and laboratory findings were entered in a standardized proforma, obtained in direct interviews and hospital records. All patients were enrolled in the follow-up system of the tertiary care hospital and given a different registration number and discharge booklet to be used in follow-up. Registered patients were enrolled only to maximize compliance with the follow-up. In the second phase of data collection, the information about hospital readmission, adherence to therapy, and follow-up practices was obtained in the outpatient records and clinical files. In cases where the participants could not be reached during the initial contact, two further contact attempts were undertaken after every one week before participants were classified as non-respondents. The commonest reasons to non-response were lost to follow-up and death. The research personnel were trained to ensure that all data collected were inputted and checked by trained research personnel to improve accuracy and consistency.

Each enrolled patient provided data covering sociodemographic characteristics, i.e., age, gender, education, occupation, income, residential area, BMI (kg/m²), and tobacco use. Clinical details encompassed the duration and type of HF, classified according to ejection fraction into HF with reduced ejection fraction (HFrEF; left ventricular ejection fraction (LVEF) <40%), mildly reduced ejection fraction (HFmrEF; LVEF 41-49%), and preserved ejection fraction (HFpEF; LVEF >50%), as well as the New York Heart Association (NYHA) functional class (Class I: no limitation of physical activity; Class II: slight limitation; Class III: marked limitation; Class IV: symptoms at rest) [15], and coexisting illnesses, including hypertension, diabetes mellitus, chronic kidney disease (CKD), coronary artery disease (CAD), anemia, and atrial fibrillation, were noted. Regular examinations included hemoglobin, creatinine, estimated glomerular filtration rate (eGFR), sodium, potassium, and N-terminal pro-B-type natriuretic peptide (NT-proBNP) levels. The information on the treatment was also discussed during the hospital discharge with the prescribed classes of medications and non-drug approaches to the management (diet, salt restriction, and fluid control). The post-discharge monitoring variables comprised adherence behavior, first week follow-up, geographical accessibility, and family support. Any non-elective hospitalization within 90 days of worsening of HF or acute decompensation or comorbid conditions was included in the unplanned re-hospitalization/readmission definition. The patients were educated on symptom identification and the value of follow-up visits.

IBM SPSS Statistics for Windows, V. 26.0 (IBM Corp., Armonk, NY, USA), was used for data analysis. Quantitative variables were summarized as mean ± SD when the distribution was normal or as median and interquartile range in case of skewed data. Numbers and percentages were used to present qualitative variables. For comparative analysis, differences between non-numerical variables were tested using the chi-squared test, while continuous measures were analyzed using the independent t-test. Covariates showing a p-value of <0.10 on univariate analysis were subsequently analyzed using multivariable logistic regression to identify independent predictors of 90-day re-hospitalization/readmission. The outcomes were presented as adjusted odds ratios (AOR) with 95% confidence intervals (CI), and a two-tailed p-value of <0.05 denoted statistical significance.

## Results

Of 300 participants, 270 (90%) completed the follow-up period of 90 days, while 17 deaths during follow-up and 13 losses to contact were recorded. The readmission rate was 28.9% (n = 78). Readmitted patients were significantly older (62.3 ± 12.5 vs 58.8 ± 13.0 years; p = 0.034), more often of lower socioeconomic status (47.4% vs 30.7%; p = 0.004), and more likely to have NYHA Class III functional limitation at discharge (62.8% vs 42.2%; p = 0.001). Comorbidities such as CKD (p < 0.001) and anemia (p = 0.001) were also significantly more common in the readmitted group. Laboratory results highlighted lower mean hemoglobin (11.0 ± 1.8 vs 11.7 ± 1.7 g/dL; p = 0.002), reduced eGFR (p < 0.001), and lower serum sodium (p < 0.001) in the readmitted group. Readmitted patients had significantly lower mean LVEF (32.1 ± 10.3% vs 37.4 ± 11.0%; p < 0.001) and higher NT-proBNP concentrations (median 3,400 (interquartile range 2,300-5,200) vs 2,300 (interquartile range 1,500-3,600) pg/mL; p < 0.001) (Table 1).

**Table 1:** Baseline demographic, clinical, and laboratory characteristics of HF patients at discharge from the hospital (n = 270) Continuous variables were expressed as mean ± SD or median (IQR) according to data distribution. The independent samples t-test was applied to compare means of normally distributed continuous variables between re-hospitalized and non-re-hospitalized patients. The Mann-Whitney U test (reported as Z statistic) was applied for non-normally distributed variables such as NT-proBNP. The chi-squared (χ²) test was used to assess associations between categorical variables. An asterisk (*) indicates statistical significance at p < 0.05, implying that the observed difference between groups was unlikely due to chance. SD: standard deviation; HF: heart failure; HFrEF: heart failure with reduced ejection fraction; HFmrEF: heart failure with mildly reduced ejection fraction; HFpEF: heart failure with preserved ejection fraction; NYHA: New York Heart Association functional class; BMI: body mass index; CKD: chronic kidney disease; COPD: chronic obstructive pulmonary disease; CAD: coronary artery disease; eGFR: estimated glomerular filtration rate; NT-proBNP: N-terminal pro-B-type natriuretic peptide; LVEF: left ventricular ejection fraction; IQR: interquartile range; mEq/L: milliequivalents per liter; pg/mL: picograms per milliliter; g/dL: grams per deciliter; kg/m²: kilograms per square meter; n (%): number and percentage of participants

Variable	Category/unit	Total (n = 270)	Re-hospitalized (n=78)	Not re-hospitalized (n = 192)	Test statistic	P-value
Sociodemographic						
Age	Years, mean ± SD	59.8 ± 12.9	62.3 ± 12.5	58.8 ± 13.0	t = 2.13	0.034*
Gender	Male, n (%)	178 (65.9)	53 (67.9)	125 (65.1)	χ² = 0.22	0.641
	Female, n (%)	92 (34.1)	25 (32.1)	67 (34.9)		
Marital status	Married, n (%)	198 (73.3)	53 (67.9)	145 (75.5)	χ² = 0.26	0.880
	Unmarried, n (%)	49 (18.1)	17 (21.8)	32 (16.7)		
	Widow(er), n (%)	23 (8.5)	8 (10.3)	15 (7.8)		
Socioeconomic status	Low, n (%)	96 (35.6)	37 (47.4)	59 (30.7)	χ² = 11.18	0.004*
	Middle, n (%)	138 (51.1)	35 (44.9)	103 (53.6)		
	High, n (%)	36 (13.3)	6 (7.7)	30 (15.6)		
Residence	Urban, n (%)	194 (71.9)	50 (64.1)	144 (75)	χ² = 1.51	0.219
	Rural, n (%)	76 (28.1)	28 (35.9)	48 (25)		
Smoking status	Never, n (%)	148 (54.8)	35 (44.9)	113 (58.9)	χ² = 0.42	0.811
	Former, n (%)	65 (24.1)	22 (28.2)	43 (22.4)		
	Current, n (%)	57 (21.1)	21 (26.9)	36 (18.8)		
BMI	kg/m², mean ± SD	26.1 ± 4.3	26.0 ± 4.4	26.1 ± 4.3	t = -0.18	0.861
Clinical and comorbidity						
Duration of HF	Years, mean ± SD	3.8 ± 2.9	4.2 ± 3.1	3.6 ± 2.8	t = 1.62	0.106
HF phenotype	HFrEF, n (%)	156 (57.8)	53 (67.9)	103 (53.6)	χ² = 5.68	0.058
	HFmrEF, n (%)	46 (17)	12 (15.4)	34 (17.7)		
	HFpEF, n (%)	68 (25.2)	13 (16.7)	55 (28.6)		
NYHA at discharge	II, n (%)	124 (45.9)	24 (30.8)	100 (52.1)	χ² = 13.12	0.001*
	III, n (%)	130 (48.1)	49 (62.8)	81 (42.2)		
	IV, n (%)	16 (5.9)	5 (6.4)	11 (5.7)		
Hypertension	Yes, n (%)	162 (60)	51 (65.4)	111 (57.8)	χ² = 1.22	0.269
	No, n (%)	108 (40)	27 (34.6)	81 (42.2)		
Diabetes mellitus	Yes, n (%)	105 (38.9)	36 (46.2)	69 (35.9)	χ² = 2.30	0.130
	No, n (%)	165 (61.1)	42 (53.8)	123 (64.1)		
CKD	Yes, n (%)	41 (15.2)	23 (29.5)	18 (9.4)	χ² = 16.95	<0.001*
	No, n (%)	229 (84.8)	55 (70.5)	174 (90.6)		
COPD	Yes, n (%)	28 (10.4)	11 (14.1)	17 (8.9)	χ² = 1.56	0.211
	No, n (%)	242 (89.6)	67 (85.9)	175 (91.1)		
Anemia	Yes, n (%)	78 (28.9)	34 (43.6)	44 (22.9)	χ² = 11.10	0.001*
	No, n (%)	192 (71.1)	44 (56.4)	148 (77.1)		
Atrial fibrillation	Yes, n (%)	49 (18.1)	18 (23.1)	31 (16.1)	χ² = 1.77	0.184
	No, n (%)	221 (81.9)	60 (76.9)	161 (83.9)		
CAD	Yes, n (%)	126 (46.7)	40 (51.3)	86 (44.8)	χ² = 0.87	0.351
	No, n (%)	144 (53.3)	38 (48.7)	106 (55.2)		
Laboratory and echocardiography						
Hemoglobin	g/dL, mean ± SD	11.5 ± 1.8	11.0 ± 1.8	11.7 ± 1.7	t = -3.08	0.002*
eGFR	mL/min/1.73 m², mean ± SD	66.2 ± 19.4	58.1 ± 17.8	69.4 ± 19.2	t = -4.56	<0.001*
Sodium	mEq/L, mean ± SD	135.6 ± 4.6	134.2 ± 4.9	136.1 ± 4.3	t = -3.23	0.001*
Potassium	mEq/L, mean ± SD	4.4 ± 0.5	4.4 ± 0.5	4.4 ± 0.5	t = 0.00	1.000
NT-proBNP	pg/mL, median (IQR)	2,500 (1,600-3,900)	3,400 (2,300-5,200)	2,300 (1,500-3,600)	Z = -4.12	<0.001*
LVEF	%, mean ± SD	36.0 ± 10.9	32.1 ± 10.3	37.4 ± 11.0	t = -3.74	<0.001*

Among post-discharge and healthcare-related factors, poor medication adherence showed a strong association with re-hospitalization. Nearly half of the readmitted patients (48.7%) were non-adherent compared with 21.4% of those not re-hospitalized (χ² = 19.01; p < 0.001). Lack of early follow-up within seven days of discharge was also significant, with 57.7% of such patients readmitted versus 42.3% with timely follow-up (χ² = 8.70; p = 0.003). Residence at a distance of ≥10 km from the hospital was linked to higher re-hospitalization (56.4% vs 43.6%; χ² = 5.91; p = 0.015). Additionally, patients lacking caregiver support had higher re-hospitalization/readmission rates (35.9% vs 21.4%; χ² = 5.96; p = 0.015) (Table 2).

**Table 2:** Treatment characteristics at discharge and post-discharge system factors among heart failure patients with and without re-hospitalization (n = 270) The independent samples t-test was applied to compare continuous variables between re-hospitalized and non-re-hospitalized groups. The chi-squared (χ²) test was used to assess associations between categorical variables such as drug use, compliance, and follow-up factors. An asterisk (*) denotes a statistically significant difference at p < 0.05, indicating that the observed variation between the groups was unlikely to have occurred by chance. SD: standard deviation; ACEi: angiotensin-converting enzyme inhibitor; ARB: angiotensin receptor blocker; MRA: mineralocorticoid receptor antagonist; SGLT-2 inhibitor: sodium-glucose co-transporter-2 inhibitor

Variable	Category/unit	Total (n = 270)	Re-hospitalized (n = 78)	Not re-hospitalized (n = 192)	Test statistic	P-value
Treatment at discharge						
Diuretic type	Loop, n (%)	212 (78.5)	65 (83.3)	147 (76.6)	χ² = 1.88	0.390
	Thiazide, n (%)	12 (4.4)	3 (3.8)	9 (4.7)		
	Combination, n (%)	46 (17)	10 (12.8)	36 (18.8)		
ACEi/ARB use	Yes, n (%)	213 (78.9)	49 (62.8)	164 (85.4)	χ² = 16.73	<0.001*
	No, n (%)	57 (21.1)	29 (37.2)	28 (14.6)		
Beta-blocker use	Yes, n (%)	235 (87)	64 (82.1)	171 (89.1)	χ² = 2.40	0.121
	No, n (%)	35 (13)	14 (17.9)	21 (10.9)		
MRA use	Yes, n (%)	161 (59.6)	41 (52.6)	120 (62.5)	χ² = 2.18	0.140
	No, n (%)	109 (40.4)	37 (47.4)	72 (37.5)		
SGLT-2 inhibitor use	Yes, n (%)	117 (43.3)	28 (35.9)	89 (46.4)	χ² = 2.50	0.114
	No, n (%)	153 (56.7)	50 (64.1)	103 (53.6)		
Index hospital stay	Days, mean ± SD	6.8 ± 3.0	7.8 ± 3.6	6.5 ± 2.8	t = 3.13	0.002*
Post-discharge and system factors						
Medication compliance	Good, n (%)	191 (70.7)	40 (51.3)	151 (78.6)	χ² = 19.01	<0.001*
	Poor, n (%)	79 (29.3)	38 (48.7)	41 (21.4)		
Early follow-up ≤7 days	Yes, n (%)	150 (55.6)	33 (42.3)	117 (60.9)	χ² = 8.70	0.003*
	No, n (%)	120 (44.4)	45 (57.7)	75 (39.1)		
Distance to facility	<10 km, n (%)	149 (55.2)	34 (43.6)	115 (59.9)	χ² = 5.91	0.015*
	≥10 km, n (%)	121 (44.8)	44 (56.4)	77 (40.1)		
Caregiver support	Yes, n (%)	201 (74.4)	50 (64.1)	151 (78.6)	χ² = 5.96	0.015*
	No, n (%)	69 (25.6)	28 (35.9)	41 (21.4)		

Multivariable regression analysis revealed that functional limitation at discharge, reflected by NYHA Class III status, was independently linked to a higher probability of 90-day re-hospitalization/readmission (AOR: 2.18; 95% CI: 1.15-4.13; p = 0.017). Other notable predictors included CKD (AOR: 2.34; 95% CI: 1.02-5.37; p = 0.044) and elevated NT-proBNP concentrations (AOR: 1.21 per 1,000 pg/mL; 95% CI: 1.07-1.37; p = 0.002). Failure to receive ACEi/ARB-based therapy (AOR: 2.87; 95% CI: 1.38-5.97; p = 0.005), non-adherence to medication (AOR: 2.95; 95% CI: 1.52-5.72; p = 0.001), and absence of early follow-up within seven days of discharge (AOR: 1.98; 95% CI: 1.05-3.73; p = 0.034) independently predicted re-hospitalization/readmission. The overall model demonstrated a satisfactory fit (Nagelkerke R² = 0.547; χ² = 8.34; p = 0.401) and strong discriminatory power (area under the curve (AUC) = 0.862; 95% CI: 0.812-0.912; sensitivity: 73.1%; specificity: 85.4%) (Table 3).

**Table 3:** Multivariable logistic regression analysis identifying independent predictors of re-hospitalization among patients with heart failure (n = 270) An asterisk (*) signifies statistical significance at p < 0.05, indicating that the variable was an independent predictor of re-hospitalization after adjustment for other factors in the regression model. OR: odds ratio; CI: confidence interval; NYHA: New York Heart Association functional class; CKD: chronic kidney disease; eGFR: estimated glomerular filtration rate; NT-proBNP: N-terminal pro-B-type natriuretic peptide; LVEF: left ventricular ejection fraction; ACEi: angiotensin-converting enzyme inhibitor; ARB: angiotensin receptor blocker; mEq/L: milliequivalents per liter; pg/mL: picograms per milliliter; km: kilometer

Variable	Reference/coding	Adjusted OR (95% CI)	P-value
Age	Per 1-year increase	1.01 (0.98-1.04)	0.532
Socioeconomic status	Middle vs low	0.61 (0.32-1.16)	0.131
Socioeconomic status	High vs low	0.44 (0.14-1.38)	0.158
NYHA	Class III vs Class II	2.18 (1.15-4.13)	0.017*
NYHA	Class IV vs Class II	1.45 (0.38-5.52)	0.587
CKD	Yes vs no	2.34 (1.02-5.37)	0.044*
Anemia	Yes vs no	1.28 (0.61-2.68)	0.512
eGFR	Per 10 mL/min/1.73 m² increase	0.88 (0.71-1.09)	0.241
Sodium	Per 1 mEq/L increase	0.95 (0.89-1.02)	0.156
NT-proBNP	Per 1000 pg/mL increase	1.21 (1.07-1.37)	0.002*
LVEF	Per 1% increase	0.97 (0.94-1.01)	0.118
ACEi/ARB	No vs yes	2.87 (1.38-5.97)	0.005*
Index hospital stay	Per 1-day increase	1.09 (0.98-1.21)	0.108
Medication compliance	Poor vs good	2.95 (1.52-5.72)	0.001*
Early follow-up (≤7 days)	No vs yes	1.98 (1.05-3.73)	0.034*
Distance to facility	≥10 km vs <10 km	1.52 (0.81-2.87)	0.194
Caregiver support	No vs yes	1.75 (0.87-3.52)	0.117

In the final regression model, six independent variables predicted readmission within 90 days of discharge. Those with NYHA Class III symptoms at discharge had more than twofold higher odds of readmission compared with patients with NYHA Class I-II symptoms (AOR: 2.18; 95% CI: 1.15-4.13; p = 0.017). The presence of CKD independently predicted readmission (AOR: 2.34; 95% CI: 1.02-5.37; p = 0.044). Additionally, each 1,000 pg/mL increase in NT-proBNP was associated with a 21% higher odds of readmission (AOR: 1.21 per 1,000 pg/mL; 95% CI: 1.07-1.37; p = 0.002). Failure to receive ACEi or ARB therapy was linked to greater re-hospitalization risk (AOR: 2.87; 95% CI: 1.38-5.97; p = 0.005). Furthermore, poor adherence to medication (AOR: 2.95; 95% CI: 1.52-5.72; p = 0.001) and lack of early clinical follow-up (AOR: 1.98; 95% CI: 1.05-3.73; p = 0.034) independently contributed to readmission. The model accounted for 54.7% of outcome variance (Nagelkerke R² = 0.547) and exhibited strong calibration (χ² = 8.34; p = 0.401) with excellent discriminative performance (AUC = 0.862).

## Discussion

This study evaluated demographic, clinical, biochemical, therapeutic, and healthcare-related predictors of 90-day unplanned re-hospitalization among patients with HF treated at a tertiary-level center. Through the analysis, it was noted that almost one-third of the patients were re-hospitalized during the follow-up period and this is indicative of the persistent issue of recurring hospitalization irrespective of modern treatment of the condition. It was identified that six parameters independently predict readmission, including discharge NYHA Class III, CKD, elevated NT-proBNP levels, lack of ACEi/ARB therapy, optimal medication adherence, and the inability to receive early post-discharge review within seven days.

The re-hospitalization rate of 28.9% reported during this research is within the limits of the large international registries. Past extensive studies in the United States have recorded readmission rates of 30-90 days as between 31% and 37%, which has indicated the global burden of recurrent hospitalization of HF [16,17]. Another study found a readmission rate of 36.6%, and about a third of them involved cardiac causes [18]. This improved readmission rate in the current cohort could be due to variation in population age, healthcare-seeking behavior, and shorter life expectancy in a lower- and middle-income environment, whereby competing non-cardiac mortality tends to reduce the follow-up period [19].

Poor NYHA functional class at discharge turned into a separate predictor of readmission risk. The odds ratio (OR) of re-hospitalization in patients who had at discharge an NYHA Class III level was more than twofold, indicating that remaining congestion and poor symptom management at discharge continued to be the primary factors that drive early recurrence. The findings are in line with those of Al-Tamimi et al., who found that an increase in the NYHA class by itself elevated the risk of 90-day readmission (OR: 2.22; 95% CI: 1.12-4.43) [20]. The same associations were reported in a prospective cohort by Wideqvist et al.: incomplete hemodynamic recovery and increased neurohormonal activation (so-called) after discharge are the hallmarks of the so-called vulnerable phase [5]. Pathophysiologically, sustained venous pressure and high filling pressure damage renal perfusion, activate the renin-angiotensin-aldosterone system (RAAS), and add to the early reoccurrence of decompensation [6]. All these processes are relevant to the reasons why functional status at discharge is one of the strongest predictors of short-term outcomes in the entire population of HF.

CKD was associated with more than a twofold higher odds of re-hospitalization in this study. This finding is consistent with Bhosale et al., who demonstrated CKD as a key predictor of 60-day readmission (OR: 3.06; 95% CI: 1.10-57.04), and Yawalkar and Gajbhiye, who likewise identified CKD as a significant determinant of readmission (OR: 2.45; p = 0.005) [7,21]. The bidirectional relationship between renal dysfunction and HF is well established, wherein impaired renal function leads to fluid retention, electrolyte imbalance, and limited tolerance to neurohormonal blockade, while reduced cardiac output exacerbates renal hypoperfusion and accelerates kidney injury. This "cardiorenal syndrome" creates a vicious cycle of congestion, diuretic resistance, and accelerated disease progression [9,11].

The NT-proBNP levels were observed to represent a considerable and independent predictor of frequent hospitalization. The increase of 1,000 pg/mL was accompanied by a 21% increase in the risk of readmission, which is consistent with other studies that have shown that above 3,000 pg/mL is an indicator of frequent hospitalization [21]. NT-proBNP is a manifestation of the synergistic interaction of the effect of stress on the ventricular wall, fluid retention, and neurohormonal activity on a physiological level. The continuing high level after discharge typically implies the failure to adequately decongest or incompletely treat cardiac dysfunction. NT-proBNP has been extensively proven as a prognostic factor in global studies, which only enhances its role as a clinical biomarker of early risk detection and maximizing treatment options [22]. The noted correlation in this cohort highlights the possible practical value in serial NT-proBNP monitoring to provide personalized treatment and prompt therapeutic change.

There was a substantial effect of therapeutic factors on readmission risk. Patients who were not put under renin-angiotensin system blockade, be it in the form of ACEis or ARBs, were almost three times more likely to be re-hospitalized. Similarly, previous reported literature have shown that guideline-directed medical therapy (GDMT) using these agents is effective in reducing morbidity and mortality in patients with HFrEF [23,24]. Lack of diuretic and beta-blocker therapy was also found by Yawalkar and Gajbhiye to be the key factor behind the frequent hospitalization, and the significance of pharmacologic optimization is also a part of the secondary prevention [21].

Poor medication adherence emerged as one of the strongest independent predictors in our study, and it elevated the rates of readmission by almost three times. Consistent with prior evidence, Sadiq et al. reported that non-adherence doubled the risk of readmission, while Wideqvist et al. observed non-adherence to medications in 42% of patients who were re-hospitalized [5,8]. Likewise, both Western and Asian studies have reported medication non-adherence as an interventable factor of early readmission [25,26]. Non-adherence diminishes neurohormonal blockade, predisposing to rapid fluid overload and recurrence of symptoms. Other factors that lead to poor adherence are behavioral and socioeconomic, such as low health literacy, polypharmacy, and high cost of medication [10]. All these findings highlight the importance of patient education programs, simplified regimens, and pre-discharge counseling by pharmacists.

Another outcome determinant was early post-discharge follow-up. Patients who missed follow-up within seven days after discharge were almost twice as likely to be re-hospitalized. This correlation supports the results of Lal et al., who also found that an absence of regular follow-up was a statistically significant predictor of 90-day readmission (p = 0.024) [11]. European and North American registries have also reported similar evidence with structured follow-up during the first week of discharge having a significant reduction of re-hospitalization and mortality [27]. Pre-emptive assessment helps to alter medications, support self-care, and identify the presence of fluid retention or drug side effects, which can be used to avoid frequent instances of decompensation. These findings support integrating multidisciplinary post-discharge interventions and telemonitoring approaches into the everyday care of HF patients, which has been previously supported by the existing research [12,13].

Other parameters were found to be associated during the univariate analysis, such as anemia, decreased LVEF, hyponatremia, and decreased eGFR. These variables were, however, not statistically significant in the multivariate model. Anemia and hyponatremia are both considered to be indicators of end-stage HF since they are indications of fluid overload and neurohormonal imbalance [11,14]. The same finding was observed by Bhosale and colleagues, who stated that increased hemoglobin and LVEF were correlated with reduced re-hospitalization risk, which indicated improved myocardial reserve and oxygen delivery. This means that the attenuation of these associations post-adjustment would signify that they have been mediated by disease severity, renal function, and medication adherence [7].

In the adjusted analysis, sociodemographic factors were not a predictor of readmission independently (age, gender, and socioeconomic background). Even though older age has a significant association with readmission in the univariate analysis, it lost its meaning when comorbidities and functional limitations were addressed. Past studies have given inconsistent findings, with some reporting younger people (below 65 years old) as at a greater risk because of the more aggressive disease and psychosocial instability [28] and others highlighting the accumulative burden of frailty and multimorbidity among the elderly [19]. These results suggest that behavioral, clinical, and care-related variables have a more powerful effect on early readmission than demographic variables.

From a pathophysiological standpoint, recurrent HF decompensation reflects a synergistic interaction between neurohormonal overactivation, hemodynamic instability, and patient-related behaviors. The constant stimulation of the RAAS and sympathetic nervous system plays a role in sodium retention, vascular remodeling, and gradual myocardial impairment. Simultaneously, low medication compliance and lack of follow-up worsen congestion and inflammation, leading to the development of a clinical deterioration cycle [6,23]. The key predictors identified in this study, such as renal impairment, high NT-proBNP, and low treatment adherence, represent downstream manifestations of these mechanisms. Addressing both biological and behavioral contributors is essential to break the cycle of repeated decompensation and reduce readmission risks successfully [6,18].

The results of the current research are clinically relevant since they indicate the necessity of early functional evaluation, pharmacologic therapy optimization, organized discharge instructions, and timely post-discharge follow-up. The distinct focus should be on the patients with high NT-proBNP levels, CKD, or inadequate adherence to the prescribed medications at the time of discharge, since they might benefit from transitional care programs. Telehealth monitoring programs combined with clinical pharmacy support and timely follow-up appointments have continued to show an advantage in enhancing adherence and decreasing repeat admissions in different international studies [12,13]. These strategies are viable and cost-effective means of enhancing outcomes, considering the unavailability of local data and the resources available in the low- and middle-income settings.

The present study has some limitations. The principal limitation is its single-center design and consecutive non-probability sampling, which can affect external validity. Absence of blinding, incomplete information on medication titration, and potential unmeasured confounders represent additional sources of bias. Caution should then be exercised in the generalization of the results to the wider populations. The study, however, provides useful context-dependent information on the problem of short-term re-hospitalization of HF with an accent on the integrated effects of clinical, behavioral, and healthcare-related factors. The findings emphasize the importance of medication compliance, timely follow-ups, and maximization of evidence-based treatment and, thus, provide practical goals to minimize recurrent hospitalizations. Despite these limitations, the study provides valuable context-specific insights into short-term HF re-hospitalization and underscores the need for integrated, multidisciplinary post-discharge care models.

## Conclusions

The current analysis indicated that the determinants of early re-hospitalization among HF patients are highly interrelated, highlighting the complexity of the association between clinical severity, comorbidity, adherence, and continuity of care. Persistent disease activity was evidenced by residual functional limitation at discharge, renal dysfunction, and elevated natriuretic peptide levels. In the meantime, the deficiencies of transitional care and the engagement with patients were reflected in poor therapy adherence and inadequate follow-ups. These findings in combination support the importance of individualized discharge planning, therapy optimization, and long-term patient and caregiver interaction. Comprehensive discharge planning, optimization of therapy, and sustained patient engagement through coordinated transitional care are essential to reduce recurrent HF hospitalizations.
